# Respiratory viral co-detection in paediatric SARS-CoV-2 infection: associations with viral co-circulation and clinical phenotypes

**DOI:** 10.1590/0074-02760260121

**Published:** 2026-08-31

**Authors:** Jayra Juliana Paiva Alves, Iago de Souza Gomes, Themis Rocha de Souza, Isabelle Cristina Clemente Santos, Pedro Henrique Ferreira Cavalcanti, Janaína Sonale Cavalcante Nogueira de Oliveira, Sônia Carmem Morais Leite, Ana Paula Ferreira Costa, Magaly Cristina Bezerra Câmara, Derley Galvão de Oliveira, Ana Helena Silva Moura, Wallace Pitanga Bezerra, Senei da Rocha Henrique, Karoline Ferreira Nascimento de Oliveira, Laiane Graziela Paulino da Costa, Paola Cristina Resende, Marcelo Ribeiro-Alves, Marilda Mendonça Siqueira, Josélio Maria Galvão de Araújo, Cristiana Couto Garcia

**Affiliations:** 1 Fundação Oswaldo Cruz-Fiocruz, Instituto Oswaldo Cruz, Programa de Pós-Graduação em Biologia Parasitária, Rio de Janeiro, RJ, Brasil; 2 Laboratório Central de Saúde Pública do Estado do Rio Grande do Norte, Natal, RN, Brasil; 3 Universidade Federal do Rio Grande do Norte, Centro de Biociências, Departamento de Microbiologia e Parasitologia, Natal, RN, Brasil; 4 Instituto de Ensino, Pesquisa e Inovação da Liga Contra o Câncer, Natal, RN, Brasil; 5 Northwestern University, Feinberg School of Medicine, Centre for Human Immunobiology, Chicago, USA; 6 Secretaria de Estado de Saúde Pública do Rio Grande do Norte, Natal, RN, Brasil; 7 Fundação Oswaldo Cruz-Fiocruz, Instituto Oswaldo Cruz, Laboratório de Vírus Respiratórios, Exantemáticos, Enterovírus e Emergências Virais, Rio de Janeiro, RJ, Brasil; 8 Fundação Oswaldo Cruz-Fiocruz, Instituto Nacional de Infectologia Evandro Chagas, Laboratório de Pesquisa Clínica em HIV, Aids, IST e Hepatites, Rio de Janeiro, RJ, Brasil; 9 Fundação Oswaldo Cruz-Fiocruz, Instituto René Rachou, Grupo Integrado de Pesquisas em Biomarcadores, Belo Horizonte, MG, Brasil

**Keywords:** SARS-CoV-2, respiratory viral co-detection, viral co-circulation, number of co-detected viruses, multiplex PCR, paediatric infection

## Abstract

**BACKGROUND:**

Respiratory viral infections in children are characterised by the co-circulation of multiple viral pathogens, but the relevance of severe acute respiratory syndrome coronavirus 2 (SARS-CoV-2) co-detection with other respiratory viruses remains unclear.

**OBJECTIVES:**

To evaluate the clinical and epidemiological implications of respiratory viral co-detection and the impact of viral diversity in paediatric SARS-CoV-2 infection.

**METHODS:**

We conducted a retrospective-prospective molecular epidemiology study including children (0-11 years) with reverse transcription-polymerase chain reaction (RT-PCR)-confirmed SARS-CoV-2 infection in Natal, northeast Brazil, between 2021 and 2024. Samples were analysed by multiplex RT-PCR for ten respiratory viruses. Associations between co-detection, the number of co-detected viruses, and clinical manifestations were assessed using multivariable logistic regression.

**FINDINGS:**

Among 545 cases, 13.2% showed viral co-detection. Co-detection was associated with dyspnoea, respiratory distress, low oxygen saturation, and diarrhoea, and inversely associated with cough. An increasing number of co-detected viruses was linked to more severe manifestations. Distinct viral combinations were associated with different clinical profiles. SARS-CoV-2 viral load and variants were not associated with co-detection.

**MAIN CONCLUSIONS:**

Respiratory viral co-detection is associated with distinct clinical and epidemiological profiles influenced by the number of co-detected viruses and viral combinations. These findings highlight the role of viral co-circulation and suggest interactions among respiratory viruses in the clinical expression of disease in children.

Respiratory viral infections are highly prevalent during childhood and represent a major cause of morbidity, hospitalisation, and mortality worldwide.^1^ ^,^ ^2^ Viruses are the primary aetiological agents of acute respiratory infections, representing a substantial clinical and economic burden globally.^3^ ^,^ ^4^ ^,^ ^5^ Severe acute respiratory syndrome coronavirus 2 (SARS-CoV-2), first identified in late 2019, rapidly spread worldwide, leading to the Coronavirus disease 19 (COVID-19) pandemic.^6^ ^,^ ^7^ ^,^ ^8^ Although the global Public Health Emergency of International Concern ended in 2023, SARS-CoV-2 continues to circulate and evolve, necessitating sustained epidemiological surveillance.^6^ ^,^ ^7^ ^,^ ^9^ ^,^ ^10^ ^,^ ^11^

In children, SARS-CoV-2 infection is generally less frequent and milder than in adults.^12^ ^,^ ^13^ ^,^ ^14^ Nevertheless, severe cases in children may occur, particularly in younger children and those with comorbidities.^15^ During the COVID-19 pandemic, diagnostic efforts were largely focused on SARS-CoV-2, resulting in reduced testing for other respiratory viruses. This may have masked the true dynamics of viral co-circulation, especially in the paediatric population, in which the clinical significance of SARS-CoV-2 co-detection with other respiratory viruses remains unclear.^16^ ^,^ ^17^ ^,^ ^18^ This study aimed to investigate patterns of respiratory viral co-detection and co-circulation in paediatric SARS-CoV-2 infection, as well as the impact of the number of co-detected viruses, and specific viral combinations on disease expression.

## MATERIALS AND METHODS

*Study design and population* - A retrospective and prospective observational study was conducted using respiratory samples from paediatric patients aged 0-11 years in Rio Grande do Norte, Brazil. These samples were referred to the Central Public Health Laboratory (LACEN-RN) for SARS-CoV-2 molecular diagnosis between January 2021 and December 2024. Nasopharyngeal swabs were collected from symptomatic patients in primary healthcare units, paediatric hospitals, and sentinel surveillance sites and processed at the State Reference Laboratory (Central Laboratory Dr Almino Fernandes, LACEN/RN). The study was approved by the institutional ethics committee (CAAE: 65128122.3.0000.5537).

*Laboratory procedures* - Nasopharyngeal samples were analysed using real-time reverse transcription polymerase chain reaction (RT-qPCR). All samples were initially tested for SARS-CoV-2 (Allplex Seegene and VR1/VR2-Biomanguinhos). Positive samples were further analysed using multiplex RT-PCR (Panels 1 and 2 of respiratory viruses, Seegene) to detect additional respiratory viruses, including adenovirus (AdV); influenza A and B (FluA and FluB); human bocavirus (HBov), human metapneumovirus (hMPV); human rhinovirus/enterovirus (HRV); parainfluenza viruses 1-3 (PIV1, PIV2, PIV3) and respiratory syncytial virus (RSV). Mono-detection was defined as SARS-CoV-2 alone, whereas co-detection was defined as the simultaneous detection of SARS-CoV-2 with at least one additional virus tested.

*Data collection* - Epidemiological data were obtained from the Laboratory Analysis Management System (GAL), and clinical data were obtained from national notification systems, including e-SUS Notifica, for influenza-like illness (ILI) cases, and SIVEP-Gripe, for hospitalised patients with severe acute respiratory infection (SARI). A total of 590 records were initially retrieved from the GAL. After excluding 45 records due to unknown co-detection status, the final dataset included 545 confirmed paediatric SARS-CoV-2 cases.

**Fig. 1:**
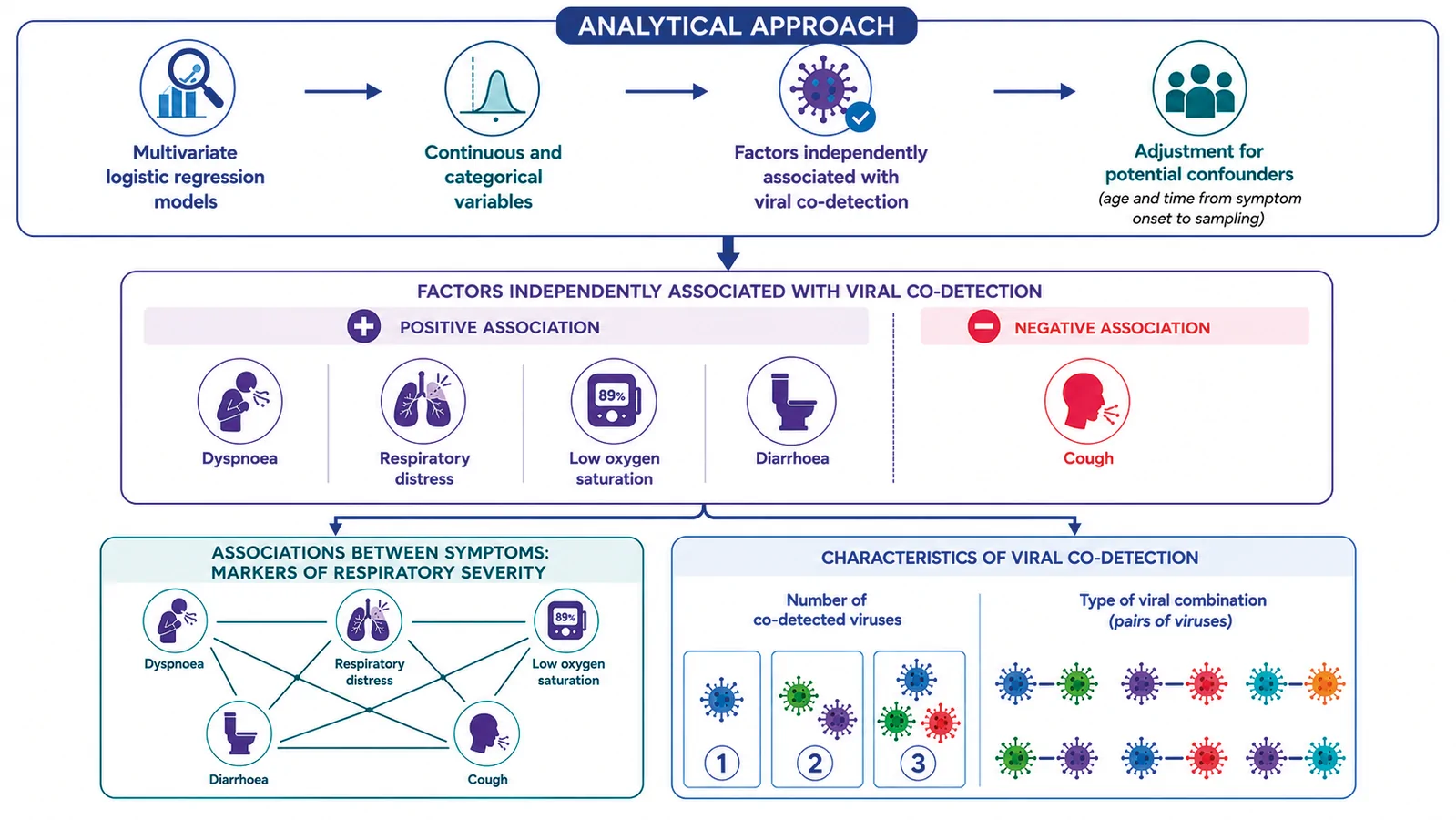
overview of the analytical strategy and main findings regarding viral co-detection among children with severe acute respiratory syndrome coronavirus 2 (SARS-CoV-2) infection from Rio Grande do Norte, northeast Brazil, between 2021 and 2024. Multivariate logistic regression analyses were performed to identify factors independently associated with viral co-detection after adjustment for age and time from symptom onset to sampling. Viral co-detection was positively associated with dyspnoea, respiratory distress, low oxygen saturation, and diarrhoea, whereas cough was negatively associated. Symptom co-occurrence patterns were examined as indicators of respiratory severity. The influence of the number of co-detected viruses and the type of viral combination on clinical presentation was also investigated. Other respiratory viruses tested: adenovirus (AdV); influenza A and B (FluA and FluB); human bocavirus (HBoV), human metapneumovirus (hMPV); human rhinovirus/enterovirus (HRV); parainfluenza viruses 1-3 (PIV1, PIV2, PIV3) and respiratory syncytial virus (RSV). Figure created by the authors and graphically refined with assistance from ChatGPT (OpenAI).

*Statistical analysis* - Descriptive analyses were performed using medians with interquartile ranges for continuous variables and proportions for categorical variables. Group comparisons employed Chi-square, Wilcoxon, and Kruskal-Wallis tests, as appropriate. Multivariate logistic regression models were constructed to identify factors independently associated with viral co-detection, adjusting for age and time from symptom onset to sampling (Fig. 1). Results were expressed as adjusted odds ratios (aOR) with 95% confidence intervals (CI). To further explore clinical relationships, additional multivariate logistic regression models were performed to evaluate associations between key respiratory symptoms, including cough, diarrhoea, dyspnoea, low oxygen saturation, and respiratory distress. Respiratory severity variables were defined according to standard clinical criteria, including dyspnoea, respiratory distress, and oxygen saturation < 95% on room air.

These models assessed whether the presence of one symptom was independently associated with the occurrence of another after adjustment for potential confounders. This approach allowed the evaluation of symptom clustering and markers of respiratory severity. Further multivariate analyses assessed the association between clinical manifestations and the cumulative number of co-detected viruses by comparing cases with two versus three detected viruses, as well as the impact of specific viral co-detection patterns, including SARS-CoV-2 combined with respiratory syncytial virus, rhinovirus/enterovirus, adenovirus, or bocavirus. These models evaluated whether symptom profiles differed according to viral number and combination.

To ensure model stability, missing data were handled using a complete-case approach. Consequently, records with unknown viral co-detection status were excluded before analysis. For other variables, values originally coded as "unknown" were treated as missing and were not imputed. Variables with insufficient variability (*i.e.*, those with less than two levels after treating "unknown" responses as missing data) were excluded from multivariate modelling to ensure model stability. Continuous variables were categorised when appropriate for analysis. Specifically, SARS-CoV-2 cycle threshold (Ct) values and time intervals (*e.g.*, days from symptom onset to sample collection) were categorised into quartiles for exploratory analysis. Age data were also processed into two categorical variables (ageYearsCat1: 1-2, 3-5, 6-11 years; ageYearsCat2: ≤ 5, > 5 years). In the multivariate logistic regression models, age and time from symptom onset to sampling were retained as continuous variables and included as adjustment confounders. Model stability was verified by assessing sparse cells and variable distribution. Bonferroni correction was applied to account for multiple testing when relevant. All analyses were conducted using R software.

*Ethics approval* - This study was performed in line with the principles of the Declaration of Helsinki and was approved by the institutional ethics committee (CAAE: 65128122.3.0000.5537).

## RESULTS

**Fig. 2:**
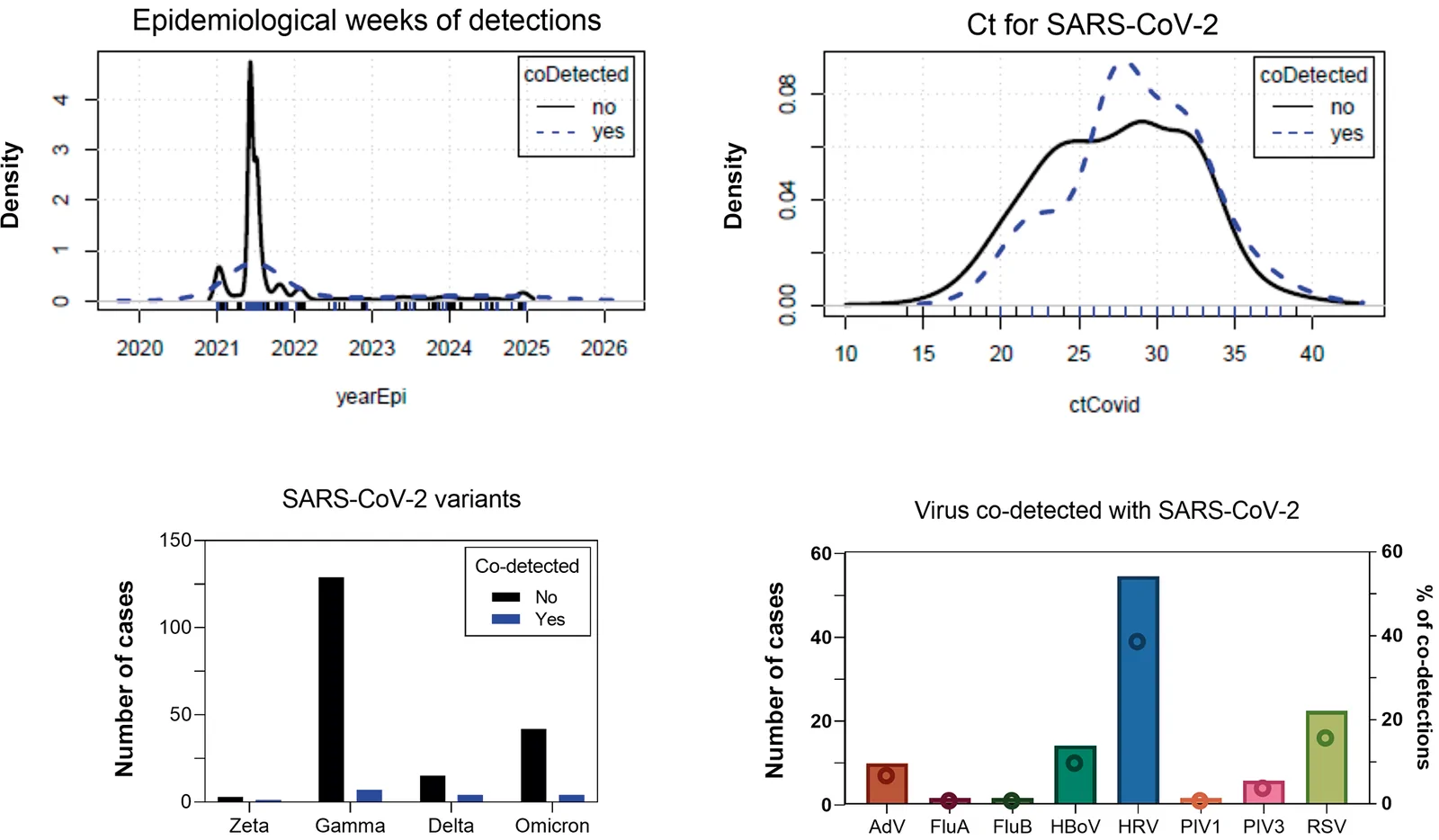
epidemiological and molecular characteristics of severe acute respiratory syndrome coronavirus 2 (SARS-CoV-2) infection among paediatric patients aged 0-11 years from Rio Grande do Norte, northeast Brazil, between 2021 and 2024: (A) Temporal distribution of respiratory virus detections by epidemiological week during the study period. (B) Distribution of cycle threshold (Ct) values from real-time reverse transcription polymerase chain reaction (RT-qPCR) assays for SARS-CoV-2–positive samples with and without co-detection. (C) Distribution of SARS-CoV-2 variants identified among sequenced samples. (D) Number of cases (circles) and percentage (bars) of co-detections between SARS-CoV-2 and the following respiratory viruses: adenovirus (AdV); influenza virus types A (FluA) and B (FluB); human bocavirus (HBoV), human rhinovirus/enterovirus (HRV); parainfluenza virus types 1 (PIV1) and 3 (PIV3) and respiratory syncytial virus (RSV).

*Epidemiological and virological characteristics* - Among 545 paediatric SARS-CoV-2 positive samples, 13.2% (n = 72) presented co-detection with one or more respiratory viruses, while 86.8% (n = 473) corresponded to single detection. Children with co-detection were significantly younger than those with single detection. Sex, race, geographic zone, and time from symptom onset to sampling were not associated with co-detection (Table I). During the sampling period, the highest SARS-CoV-2 prevalence was observed in 2021, with a four-week shift in the co-detection cases (Fig. 2A). Co-detected cases showed a higher proportion of notifications for SARI and a higher frequency of nosocomial infection, although the latter occurred at a low overall frequency.

**TABLE I:** Epidemiological and laboratory features of severe acute respiratory syndrome coronavirus 2 (SARS-CoV-2) single detection and co-detection with other respiratory viruses in paediatric patients aged 0-11 years in Rio Grande do Norte, northeast Brazil, between 2021 and 2024

			SARS-CoV-2 co-detection		
Features	Levels	Overall (n = 545)	No (%)	Yes (%)	p-value
Sex	Female	254 (46.6)	221 (46.7)	33 (45.8)	0.989
	Male	291 (53.4)	252 (53.3)	39 (54.2)	
Age	Years	6 (IQR = 8)	7 (IQR = 7)	2.5 (IQR = 6.25)	**< 0.001**
Self-reported race	White	171 (31.4)	149 (31.5)	22 (30.6)	0.08
	Black	6 (1.1)	5 (1.1)	1 (1.4)	
	Asian	32 (5.9)	31 (6.6)	1 (1.4)	
	Brown	278 (51)	233 (49.3)	45 (62.5)	
	Indigenous	0 (0)	0 (0)	0 (0)	
Zone	Urban	292 (53.6)	253 (53.5)	39 (54.2)	0.532
	Rural	83 (15.2)	75 (15.9)	8 (11.1)	
Epidemiological week of sample collection	Year. Week	2021. 48 (IQR = 0.35)	2021. 48 (IQR = 0.19)	2021. 52 (IQR = 1.5)	**0.036**
Notification type	Flu Like	484 (88.8)	430 (90.9)	54 (75)	**< 0.001**
	SARS	61 (11.2)	43 (9.1)	18 (25)	
Days from symptom to sample collection	Days	4 (IQR = 2)	4 (IQR = 2)	4 (IQR = 2.25)	0.232
Nosocomial transmission	No	52 (9.5)	36 (7.6)	16 (22.2)	**< 0.001**
	Yes	11 (2)	8 (1.7)	3 (4.2)	

Data are presented as n (%) or median (IQR - interquartile range). Percentages may not sum to 100% due to missing data. P-values were calculated using the χ² or Fisher's exact test for categorical variables and the Mann-Whitney U test for continuous variables. Other respiratory viruses: adenovirus (AdV); influenza A and B (FluA and FluB); human bocavirus (HBoV), human metapneumovirus (hMPV); human rhinovirus/enterovirus (HRV); parainfluenza viruses 1-3 (PIV1, PIV2, PIV3); and respiratory syncytial virus (RSV).

SARS-CoV-2 Ct values did not differ between groups, indicating similar viral load (Fig. 2B). No association was observed between SARS-CoV-2 lineages and co-detection. Among the SARS-CoV-2 sequenced lineages, the most frequent were lineages of the variant of concern (VOC) Gamma (P.1/P.1.), followed by VOC Omicron (B.1.1.529/BA), VOC Delta (B.1.617/AY), and Variant of Interest (VOI) Zeta (P.2) (Fig. 2C).

Rhinovirus/enterovirus was the most frequently co-detected viral group, followed by respiratory syncytial virus, bocavirus, adenovirus, and parainfluenza virus type 3. Influenza A, influenza B, and parainfluenza type 1 were rarely detected (Fig. 2D). Most co-detected samples contained two viruses (90.3%), whereas a smaller proportion showed triple detection (9.7%).

**TABLE II:** Clinical characteristics and disease severity of paediatric patients aged 0-11 years according to severe acute respiratory syndrome coronavirus 2 (SARS-CoV-2) detection status. The study was conducted between 2021 and 2024 in Rio Grande do Norte, northeast Brazil

				SARS-CoV-2 co-detection (%)		
Features		Levels	Overall (%)	No (%)	Yes (%)	p-value
Symptoms	Fever	No	167 (30.7)	149 (31.5)	18 (25.4)	0.52
		Yes	275 (50.6)	235 (49.7)	40 (56.3)	
	Cough	No	187 (34.3)	179 (37.8)	8 (11.1)	**< 0.001**
		Yes	254 (46.6)	205 (43.3)	49 (68.1)	
	Dyspnoea	No	370 (67.9)	333 (70.4)	37 (51.4)	**< 0.001**
		Yes	69 (12.7)	48 (10.1)	21 (29.2)	
	Respiratory distress	No	399 (73.2)	356 (75.3)	43 (59.7)	**< 0.001**
		Yes	43 (7.9)	28 (5.9)	15 (20.8)	
	Diarrhoea	No	411 (75.4)	362 (76.5)	49 (68.1)	**0.025**
		Yes	31 (5.7)	22 (4.7)	9 (12.5)	
	Vomiting	No	415 (76.1)	364 (77)	51 (70.8)	**0.025**
		Yes	26 (4.8)	18 (3.8)	8 (11.1)	
	Low oxygen saturation	No	423 (77.6)	373 (78.9)	50 (69.4)	**0.001**
		Yes	19 (3.5)	11 (2.3)	8 (11.1)	
	Asymptomatic	No	432 (79.3)	373 (78.9)	59 (81.9)	0.667
		Yes	16 (2.9)	15 (3.2)	1 (1.4)	
Severity	Hospitalised	No	1 (0.2)	1 (0.2)	0 (0)	**< 0.001**
		Yes	61 (11.2)	43 (9.1)	18 (25)	
	X-ray result	Normal	6 (1.1%)	4 (0.8%)	2 (2.8%)	**< 0.001**
		Interstitial infiltrates	15 (2.8%)	10 (2.1%)	5 (6.9%)	
		Consolidation	2 (0.4%)	0 (0%)	2 (2.8%)	
		Other	20 (3.7%)	14 (3%)	6 (8.3%)	
		Not performed	6 (1.1%)	3 (0.6%)	3 (4.2%)	
	ICU	No	42 (7.7)	30 (6.3)	12 (16.7)	**< 0.001**
		Yes	17 (3.1)	11 (2.3)	6 (8.3)	
	Ventilatory support	Invasive	7 (1.3)	3 (0.6)	4 (5.6)	**< 0.001**
		Non-invasive	21 (3.9)	14 (3)	7 (9.7)	
		None	29 (5.3)	22 (4.7)	7 (9.7)	
	Outcome	Recovered	59 (10.8)	46 (9.7)	13 (18.1)	0.068
		Death	2 (0.4)	1 (0.2)	1 (1.4)	
		Death from another cause	1 (0.2)	1 (0.2)	0 (0)	
Vaccine/Treatment	Vaccinated against Covid-19	No	520 (95.4)	450 (95.1)	70 (97.2)	0.461
		Yes	15 (2.8)	13 (2.7)	2 (2.8)	
	Antiviral treatment	No	56 (10.3)	40 (8.5)	16 (22.2)	**< 0.001**
		Yes	8 (1.5)	4 (0.8)	4 (5.6)	
Comorbidity	Any risk factor	No	0 (0)	0 (0)	0 (0)	0.162
		Yes	36 (6.6)	28 (5.9)	8 (11.1)	
	Asthma	No	17 (3.1)	12 (2.5)	5 (6.9)	0.116
		Yes	11 (2)	9 (1.9)	2 (2.8)	
	Neurological disease	No	11 (2)	6 (1.3)	5 (6.9)	**0.005**
		Yes	10 (1.8)	8 (1.7)	2 (2.8)	
	Immuno-suppression	No	18 (3.3)	12 (2.5)	6 (8.3)	**0.033**
		Yes	5 (0.9)	4 (0.8)	1 (1.4)	
	Cardiopathy	No	20 (3.7)	15 (3.2)	5 (6.9)	**< 0.001**
		Yes	2 (0.4)	0 (0)	2 (2.8)	
	Haematological disease	No	21 (3.9)	15 (3.2)	6 (8.3)	**0.004**
		Yes	1 (0.2)	0 (0)	1 (1.4)	
	Obesity	No	21 (3.9)	14 (3)	7 (9.7)	**0.001**
		Yes	1 (0.2)	0 (0)	1 (1.4)	
	Pulmonary disease	No	19 (3.5)	12 (2.5)	7 (9.7)	**0.008**
		Yes	1 (0.2)	1 (0.2)	0 (0)	
	Renal disease	No	21 (3.9)	14 (3)	7 (9.7)	**0.02**
		Yes	1 (0.2)	1 (0.2)	0 (0)	

Data are presented as n (%). Percentages may not sum to 100% due to missing data. P-values were calculated using the χ² or Fisher's exact test. ICU: intensive care unit. Other respiratory viruses: adenovirus (AdV); influenza A and B (FluA and FluB); human bocavirus (HBoV), human metapneumovirus (hMPV); human rhinovirus/enterovirus (HRV); parainfluenza viruses 1-3 (PIV1, PIV2, PIV3); and respiratory syncytial virus (RSV).

*Clinical characteristics* - The most frequent symptoms are listed in Table II, in descending order: fever, cough, rhinorrhoea, sore throat, and headache were reported by at least 22.4% of patients. Co-detected patients presented higher frequencies of respiratory symptoms, particularly cough, dyspnoea, respiratory distress, and low oxygen saturation. Gastrointestinal symptoms, especially diarrhoea and vomiting, were also more frequent in co-detection.

Markers of severity were more common among co-detected cases, including higher rates of hospitalisation, intensive care unit (ICU) admission, the need for invasive and non-invasive ventilatory support, and abnormal radiographic findings. Mortality was rare and similar between the groups.

Vaccination status for both COVID-19 and influenza was not associated with co-detection. The use of antiviral drugs, mainly oseltamivir phosphate, was more prevalent in the co-detected group. Only 36 (6.6%) patients had a positive record for comorbidities. Some comorbidities were more frequent among co-detected patients, in descending order: neurological diseases, immunosuppression, cardiopathy, haematological diseases, obesity, pulmonary diseases, and renal diseases, although the absolute numbers were small.

**Fig. 3:**
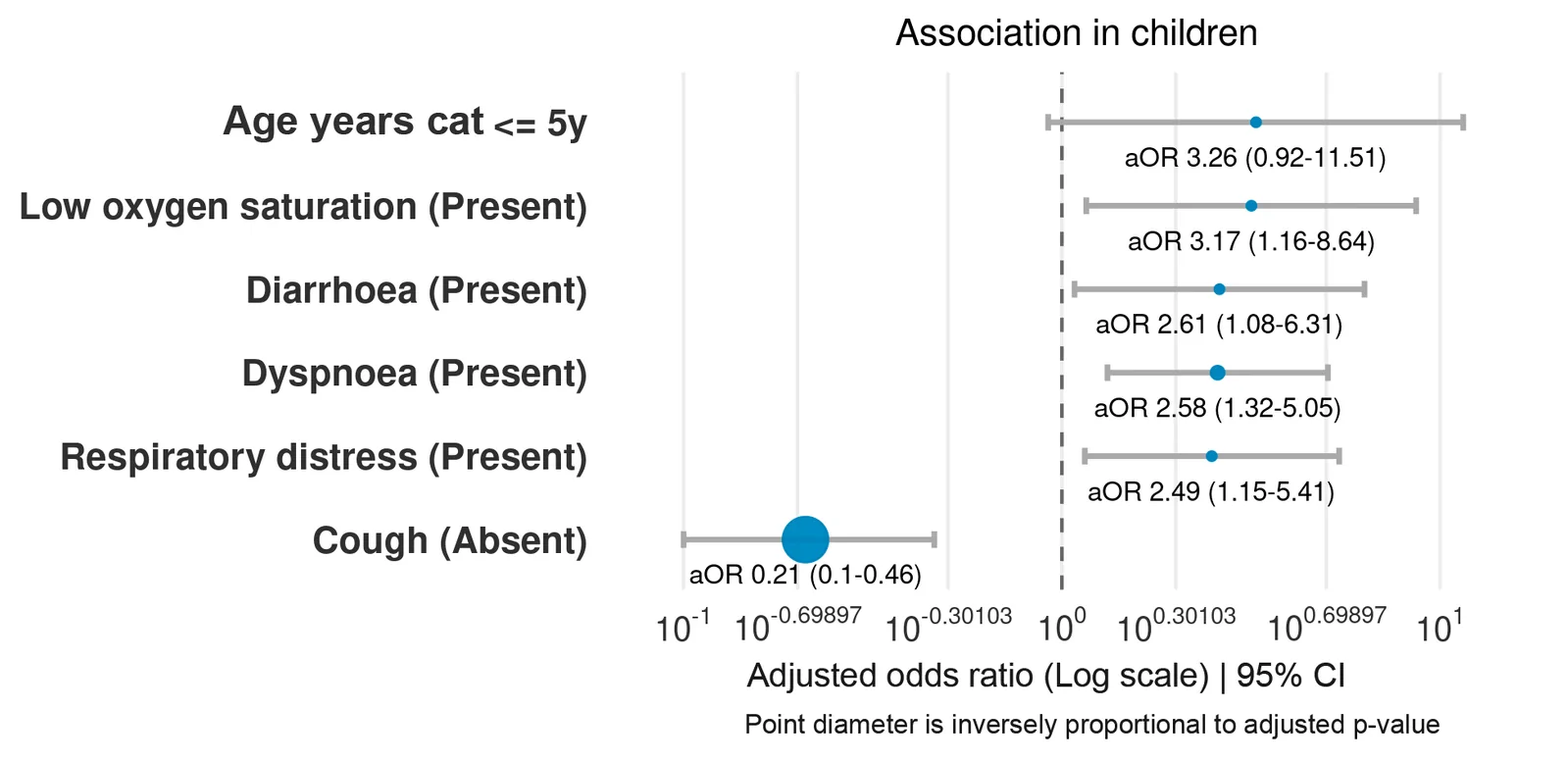
forest plot representing the adjusted odds ratio (aOR) of viral co-detection [severe acute respiratory syndrome coronavirus 2 (SARS-CoV-2) and other respiratory viruses] according to age and clinical symptoms among paediatric patients aged 0-11 years from Rio Grande do Norte, northeast Brazil, between 2021 and 2024. Other respiratory viruses tested: adenovirus (AdV); influenza A and B (FluA and FluB); human bocavirus (HBoV), human metapneumovirus (hMPV); human rhinovirus/enterovirus (HRV); parainfluenza viruses 1-3 (PIV1, PIV2, PIV3) and respiratory syncytial virus (RSV).

*Multivariate analysis* - After adjustment for key confounders, dyspnoea, respiratory distress, low oxygen saturation, and diarrhoea were independently associated with viral co-detection, each conferring an approximately two- to three-fold increased likelihood (Fig. 3). Younger age (≤ 5 years) showed a trend toward increased co-detection but did not reach statistical significance (ageYearsCat2: ≤ 5 years versus > 5 years; p = 0.066). Within the first age category, 1-2, 3-5, 6-11 years, the younger groups showed lower statistical significance (Age 1-2 p = 0.09; 3-5 p = 0.09). In contrast, cough was associated with a lower probability of co-detection (Fig. 3).

Subsequently, a symptom-to-symptom multivariate analysis demonstrated strong associations between different symptoms (Table III). Dyspnoea and respiratory distress were independently associated with cough, and respiratory distress showed a strong association with dyspnoea, reflecting the clustering of respiratory severity markers. Diarrhoea was independently associated with respiratory distress and low oxygen saturation, suggesting an overlap between gastrointestinal involvement and respiratory severity.

**TABLE III:** Multivariable analysis of symptom-to-symptom associations in severe acute respiratory syndrome coronavirus 2 (SARS-CoV-2) infection in paediatric patients aged 0-11 years in Rio Grande do Norte, northeast Brazil, between 2021 and 2024

		Symptom			
Feature	Level	Cough Absent (%)	Cough Present (%)	aOR (95%CI)	p-value
Dyspnoea	Absent	177 (95.16)	193 (76.59)	Ref.	Ref.
	Present	9 (4.84)	59 (23.41)	5.37 (2.52-11.43)	**0.00001**
Respiratory distress	Absent	183 (97.86)	216 (85.04)	Ref.	Ref.
	Present	4 (2.14)	38 (14.96)	7.07 (2.39-20.88)	**0.0004**
		Dyspnoea Absent (%)	Dyspnoea Present (%)		
Respiratory distress	Absent	366 (98.92)	30 (43.48)	Ref.	Ref.
	Present	4 (1.08)	39 (56.52)	74.28 (23.84-231.45)	**1.09 e-13**
		Diarrhoea Absent (%)	Diarrhoea Present (%)		
Respiratory distress	Absent	379 (92.21)	20 (64.52)	Ref.	Ref.
	Present	32 (7.79)	11 (35.48)	4.58 (1.73-12.11)	**0.002**
Low oxygen saturatio	Absent	399 (97.08)	24 (77.42)	Ref.	Ref.
	Present	12 (2.92)	7 (22.58)	7.05 (2.35-21.13)	**0.00049**

Each block represents a separate multivariable logistic regression model. aOR: odds ratios are adjusted for age and the number of days from symptom onset to sample collection; CI: confidence interval.

*Impact of the number of co-detected viruses* - A multivariate analysis was performed to test the cumulative number of co-detected viruses by comparing cases with two versus three detected viruses (Table IV). Increasing viral numbers influenced clinical presentation. Detection of two viruses was associated with an increased likelihood of dyspnoea, while triple viral detection markedly increased the risk of dyspnoea and diarrhoea. Only triple detection was associated with a higher probability of respiratory distress and low oxygen saturation (data not shown), indicating a potential cumulative effect of the number of co-detected viruses on disease severity, although these findings should be interpreted with caution due to the small sample size.

**TABLE IV:** Multivariable analysis of the association between the number of co-detected viruses and selected clinical manifestations in paediatric patients aged 0-11 years in Rio Grande do Norte, northeast Brazil, between 2021 and 2024

		Dyspnoea				Diarrhoea			
Feature	Level	Absent (%)	Present (%)	aOR (95%CI)	p-value	Absent (%)	Present (%)	aOR (95%CI)	p-value
Virus count	1	333 (90)	48 (69.57)			362 (88.08)	22 (70.97)		
	2	35 (9.46)	16 (23.19)	2.06 (1.01-4.22)	**0.047**	45 (10.95)	6 (19.35)	1.83 (0.67-4.95)	0.236
	3	2 (0.54)	5 (7.25)	14.59 (2.33-91.24)	**0.008**	4 (0.97)	3 (9.68)	13.34 (2.6-68.41)	**0.004**

Adjusted odds ratios (aORs) are presented; CI: confidence interval. Models were adjusted for age and the number of days from symptom onset to sample collection. A virus count of 1 (single detection) was used as the reference category. Respiratory viruses tested: adenovirus (AdV); influenza A and B (FluA and FluB); human bocavirus (HBoV), human metapneumovirus (hMPV); human rhinovirus/enterovirus (HRV); parainfluenza viruses 1-3 (PIV1, PIV2, PIV3); respiratory syncytial virus (RSV); and severe acute respiratory syndrome coronavirus 2 (SARS-CoV-2).

**TABLE V:** Multivariable analysis of specific viral co-detections and clinical manifestations in paediatric patients aged 0-11 years in Rio Grande do Norte, northeast Brazil, between 2021 and 2024

		Dyspnoea				Diarrhoea				Cough			
Feature	Level	Absent (%)	Present (%)	aOR (95%CI)	p-value	Absent (%)	Present (%)	aOR (95%CI)	p-value	Absent (%)	Present (%)	aOR (95%CI)	p-value
Co-Detection RSV x SARS-CoV-2	SC2	333 (90)	48 (69.57)			362 (88.08)	22 (70.97)			179 (46.61)	205 (53.39)		
	RSV/SC2	11 (2.97)	3 (4.35)	0.87 (0.22-3.39)	0.836	13 (3.16)	1 (3.23)	0.89 (0.11-7.43)	0.914	0 (0)	14 (100)	NC	NC
	SC2/OVR except RSV	26 (7.03)	18 (26.09)	3.66 (1.76-7.62)	**0.001**	36 (8.76)	8 (25.81)	3.26 (1.3-8.17)	**0.023**	8 (18.60)	35 (81.40)	NC	NC
Co-Detection HRV x SARS-CoV-2	SC2	333 (90)	48 (69.57)			362 (88.08)	22 (70.97)			179 (95.72)	205 (80.71)		
	HRV/SC2	18 (4.86)	16 (23.19)	4.7 (2.1-10.52)	**0.0003**	28 (6.81)	6 (19.35)	3.2 (1.15-8.92)	0.052	5 (2.67)	28 (11.02)	4.48 (1.68-11.95)	**0.005**
	SC2/OVR except HRV	19 (5.14)	5 (7.25)	1.01 (0.34-3)	0.984	21 (5.11)	3 (9.68)	1.82 (0.48-6.86)	0.377	3 (1.6)	21 (8.27)	5.46 (1.59-18.77)	**0.007**
Co-Detection AdV x SARS-CoV-2	SC2	333 (90)	48 (69.57)			362 (88.08)	22 (70.97)			179 (95.72)	205 (80.71)		
	AdV/SC2	2 (0.54)	1 (1.45)	5 (0.35-71.11)	0.235	1 (0.24)	2 (6.45)	49.26 (4.07-595.74)	**0.004**	1 (0.53)	2 (0.79)	1.81 (0.16-20.29)	0.631
	SC2/OVR except AdV	35 (9.46)	20 (28.99)	2.54 (1.29-5.01)	**0.014**	48 (11.68)	7 (22.58)	1.92 (0.74-4.96)	0.179	7 (3.74)	47 (18.5)	5.28 (2.31-12.11)	**0.0002**
Co-Detection HBOV x SARS-CoV-2	SC2	333 (90)	48 (69.57)			362 (88.08)	22 (70.97)			179 (95.72)	205 (80.71)		
	HboV/SC2	7 (1.89)	2 (2.9)	1.47 (0.27-7.99)	0.655	6 (1.46)	3 (9.68)	7.67 (1.73-34.11)	**0.015**	2 (1.07)	6 (2.36)	2.52 (0.5-12.7)	0.264
	SC2/OVR except HboV	30 (8.11)	19 (27.54)	2.87 (1.42-5.8)	**0.007**	43 (10.46)	6 (19.35)	1.89 (0.69-5.14)	0.215	6 (3.21)	43 (16.93)	5.63 (2.32-13.7)	**0.0003**

Adjusted odds ratios (aORs) are presented; CI: confidence interval. Each block represents a separate multivariable logistic regression model, adjusted for age and the number of days from symptom onset to sample collection. Single detection of severe acute respiratory syndrome coronavirus 2 (SARS-CoV-2) (SC2) was used as the reference category. NC stands for not calculable, because the absence of cough was not observed in the RSV/SC2 group. OVR stands for other respiratory viruses tested: adenovirus (AdV); influenza A and B (FluA and FluB); human bocavirus (HBoV), human metapneumovirus (hMPV); human rhinovirus/enterovirus (HRV); parainfluenza viruses 1-3 (PIV1, PIV2, PIV3); and respiratory syncytial virus (RSV).

*Viral combination and clinical phenotypes* - To investigate the association between clinical manifestations and the co-infection of SARS-CoV-2 with specific respiratory viruses (RSV, HRV, AdV, and HBoV), another multivariate analysis was performed. Distinct viral co-detection patterns were associated with a distinct symptomatology profile. RSV and SARS-CoV-2 co-detection was not independently associated with specific symptoms. In contrast, SARS-CoV-2 combined with other respiratory viruses (except for RSV) was associated with a three-fold higher likelihood of dyspnoea and diarrhoea. Patients with HRV/SARS-CoV-2 co-detection had a higher likelihood of cough and severe symptoms such as dyspnoea. AdV/SARS-CoV-2 or HBoV/SARS-CoV-2 co-detections were associated with a 49.26-fold and 7.67-fold higher risk of diarrhoea, respectively, suggesting gastrointestinal involvement. These findings indicate that specific combinations of SARS-CoV-2 with other respiratory viruses could be clinically distinguished (Table V).

## DISCUSSION

Co-detection of SARS-CoV-2 with other respiratory viruses, although little explored during the pandemic period, has received greater attention in the post-pandemic era, aiming to elucidate both the epidemiological aspects and the clinical implications of these associated infections. Despite this progress, knowledge about the co-detection of SARS-CoV-2 and other respiratory viruses in the paediatric population remains limited, highlighting the need for more in-depth investigations in this age group. This study demonstrates that respiratory viral co-detection in paediatric SARS-CoV-2 infection reflects the co-circulation of multiple respiratory viruses and is associated with distinct clinical and epidemiological profiles influenced by the number of co-detected viruses, and specific viral combinations (Fig. 1).

Among the co-detections, the viruses with the highest co-detection rate with SARS-CoV-2 were HRV (54.2%), RSV (22.2%), HBov (13.9%) and AdV (9.7%), while those with the lowest co-detection rate were PIV3 (5.6%); FluA (1.4%) and FluB (1.4%); and PIV1 (1.4%). This shows that co-infections mainly involve classic paediatric respiratory viruses, especially RSV and HRV, which have been observed in other cohorts.^19^ ^,^ ^20^ Other studies have found that, in the paediatric population, influenza A (67.6%) was the most frequent virus in co-detections with SARS-CoV-2, followed by RSV (28.3%) and influenza B (4.1%). These studies also observed a higher number of co-detections in the 0-7-year age group among paediatric samples.^21^ In this study, younger children showed a trend toward higher co-detection, which is biologically plausible given immune immaturity and greater exposure to multiple pathogens in early childhood. Although this association did not reach statistical significance after adjustment, the direction and magnitude of the effect support a possible role of age in susceptibility to viral co-detection. Several studies have demonstrated a positive association between respiratory viral co-infections and children younger than 4 years.^22^ ^,^ ^23^ ^,^ ^24^ Behavioural aspects specific to this age group, such as lower adherence to preventive practices and interaction in educational environments, intensify this risk, including for SARS-CoV-2.^25^

Specific clinical symptoms were associated with co-detection. The absence of cough reduced the risk of co-detection, suggesting that cough may be an important clinical marker for distinguishing single respiratory viral infections from multiple infections. On the other hand, cough was strongly associated with clinical signs of respiratory severity, including dyspnoea and respiratory distress. The magnitude of the observed associations suggests that cough may act as an important clinical marker of pulmonary impairment or lower airway involvement in symptomatic patients. Several symptoms indicative of disease severity, which are strongly associated with the outcome, are also included among the case-definition criteria for SARI established by both the Brazilian Ministry of Health and the World Health Organisation (WHO).^26^ Recent studies demonstrate that coughing is associated with increased airway inflammation and respiratory severity, and identify coughing as a marker of pulmonary impairment in COVID-19.^27^ ^,^ ^28^ ^,^ ^29^ ^,^ ^30^ A systematic review and meta-analysis reported a higher frequency of dyspnoea and a higher risk of mortality in patients with co-infection with SARS-CoV-2 and other respiratory viruses, compared to patients mono-infected with SARS-CoV-2.^25^ These findings support the hypothesis that isolated infections tend to have less systemic inflammation, and the simultaneous involvement of multiple viruses tends to increase this process, contributing to the risk of lung damage that might be observed clinically as dyspnoea, respiratory distress, and low oxygen saturation.

The clustering of respiratory symptoms observed in the multivariate analysis highlights the close clinical relationship between dyspnoea, respiratory distress, and hypoxemia as markers of respiratory severity. The strong association between respiratory distress and dyspnoea reinforces the conceptual and clinical proximity between these two outcomes, since dyspnoea is often the subjective manifestation of deterioration in respiratory function that, in more advanced stages, can progress to respiratory failure. However, the very large effect size should be interpreted with caution, as it likely reflects the low number of events and possibly wide confidence intervals. In such situations, estimates may become unstable and overestimate the true magnitude of the association. Taken together, these findings highlight that the combination of respiratory symptoms, rather than isolated symptoms, may be more informative in identifying patients at higher risk of severity, contributing to clinical assessment and decision-making in surveillance and care settings.

Importantly, both the cumulative number of co-detected viruses and specific viral combinations influenced clinical presentation. Detection of multiple viruses, particularly triple detection, was a risk factor for the development of severe symptoms such as dyspnoea or gastrointestinal symptoms, particularly diarrhoea. This finding corroborates previous studies indicating that infection with two or more respiratory viruses can increase disease severity and is associated with a higher risk of ICU admission compared with single infections.^31^ ^,^ ^32^ Thus, the cumulative impact of viral diversity contributes to worse clinical outcomes. Distinct viral combinations showed different clinical phenotypes. Co-detection of HRV and SARS-CoV-2 showed a significantly increased likelihood of cough and dyspnoea, suggesting that these viruses may interact to exacerbate respiratory inflammation, potentially serving as a key driver of severity in co-detected cases. HRV is traditionally recognised for its ability to induce bronchial hyperreactivity and mucosal irritation, which may explain the intensification of symptoms when present concomitantly with SARS-CoV-2.^33^ Furthermore, rhinovirus infection has been associated with hospitalisation during the first year of life and is recognised as a predictor of recurrent wheezing, a clinical manifestation related to airway narrowing and/or bronchial inflammation, and has been implicated in up to 50% of asthma exacerbations. Evidence suggests that these associations may be more related to age and greater host susceptibility to rhinovirus infection, including genetic factors, rather than to the greater intrinsic virulence of the virus.^31^ ^,^ ^34^ ^,^ ^35^

On the other hand, adenovirus and bocavirus, when co-detected with SARS-CoV-2, showed a significant association with diarrhoea. Gastrointestinal symptoms have been frequently associated with viral co-detection with SARS-CoV-2, especially involving bocavirus and adenovirus.^36^ Gastrointestinal symptoms are commonly described in SARS-CoV-2 infections.^37^ However, these manifestations may result from co-infections with respiratory and enteric viruses. The overlap of symptoms can make clinical interpretation difficult and potentially lead to inaccurate diagnostic assessments. Therefore, the differential diagnosis of these viruses is necessary.

Neither specific SARS-CoV-2 variants nor viral loads were significantly associated with co-detection. Despite this lack of association, the circulating variants were predominantly Gamma, followed by Omicron and Delta, in descending order of frequency. Both results were similar to those reported in the study by Bastard et al.,^38^ in which no association was observed between either variables and the presence of co-detection. Analysis of the variants revealed that co-detection cases occurred predominantly during the circulation period of the Delta and, especially, Omicron variants, as identified by sequencing. This temporal pattern coincides with the resumption of in-person school activities and the progressive relaxation of mitigation measures, suggesting that the increase in viral diversity in the community environment may have favoured the occurrence of co-detections.

In summary, respiratory viral co-detection in paediatric SARS-CoV-2 infection is relevant within the respiratory viral landscape rather than being a rare diagnostic finding. Our results indicate that both the number of co-detected viruses, and the specific co-detected virus contribute to the clinical presentation, reflecting a complex dynamic interplay between co-circulating viruses and host responses. Clinically, these findings emphasise the value of multiplex molecular testing not only for aetiological diagnosis but also for improved characterisation of viral circulation and clinical risk stratification in paediatric populations to support more targeted clinical management. Although the cross-sectional design and limited subgroup sizes warrant cautious interpretation, this study provides consistent evidence that respiratory viral co-detection in paediatric SARS-CoV-2 infection is not incidental but represents a clinically relevant condition associated with increased disease severity.

This study has several limitations that should be acknowledged. First, the investigation was restricted to a single state in the north-eastern Brazilian region, which may limit the generalisability of the findings to other geographic regions with distinct epidemiological profiles, seasonal patterns, or healthcare access. Second, the statistical analysis relied on a complete-case approach, excluding records with missing data on co-detection status or clinical variables. While this strategy was necessary to ensure model stability, it may introduce selection bias if the data were not missing completely at random.^39^ Finally, the number of cases with triple viral detection was relatively low, which limited the statistical power and precision of the estimates regarding the impact of a high cumulative number of co-detected viruses; consequently, these specific observations should be interpreted with caution and warrant confirmation in larger, multicentre cohorts.

In conclusion, SARS-CoV-2 co-detection with other respiratory viruses, although relatively uncommon, is clinically relevant in paediatric populations and is associated with increased respiratory severity. Both the cumulative number of co-detected viruses and specific viral combinations influenced disease manifestations, indicating that viral interactions may modulate clinical outcomes beyond single-virus infection. These findings support the concept that interactions among co-circulating respiratory viruses may contribute to heterogeneous disease expression in paediatric populations.

## Data Availability

After publication the data will be available on demand to corresponding author.
