# Supplementary material for: Respiratory viral co-detection in paediatric SARS-CoV-2 infection: associations with viral co-circulation and clinical phenotypes
Source: Mem Inst Oswaldo Cruz. 2026 Aug 31;121:e260121. doi: 10.1590/0074-02760260121 (PMC13528231; doi:10.1590/0074-02760260121)
Supplement: Peer review history

# Reviewer report: FIRST REVIEW ROUND - REVIEWERS' COMMENTS

This paper presents interesting findings and makes a strong contribution to the understanding of the role of respiratory virus co-detection with SARS-CoV-2 on disease severity in the pediatric population. The objectives are clearly stated, the manuscript is well-structured and well-written, and the methodology is well-designed. However, a few clarifications are needed prior to publication. My comments and suggestions are as follows:

1. The authors mention removing variables with excessive missing data. Could you please specify the threshold used to justify variable exclusion? I also suggest including a brief description of the total number of records collected and the number excluded after data cleaning, to improve transparency and reproducibility.

2. Which continuous variables were categorized for the analysis? Additionally, regarding the adjustment for age groups, which age intervals were considered? Please specify these details in the Methods section.

3. On page 15, line 44: several of the severity symptoms described as closely associated are also used as classification criteria for SARI cases by the Brazilian Ministry of Health and the WHO. I believe that acknowledging this in the Discussion would enrich the contextualization of the findings.

4. I also suggest providing more detail in the figure and table legends, including the study setting and the period of data collection.

## REVIEWER #2

The authors of the manuscript "Respiratory viral co-detection and viral burden in pediatric SARS-CoV-2 infection: associations with viral co-circulation and clinical phenotypes" (ID: MIOC-2026-0121), present a study evaluating the frequency of respiratory virus coinfections with SARS-CoV-2 and the association of such coinfections with different clinical manifestations. The study was conducted during the period from January 2021 to December 2024.

The results are relevant, since, as indicated by the authors, during the COVID-19 pandemic surveillance of respiratory viruses other than SARS-CoV-2 was restricted in some countries, generating important epidemiological and clinical knowledge gaps with respect to cocirculation of respiratory viruses, the frequency of coinfections and the impact on disease severity. In this context, it is sometimes assumed that infections involving more than one respiratory pathogen produce more severe disease. However, this may not be the case because of viral interference.

The results presented by the authors may be useful for clinical management and patient care.

Overall, the manuscript is well written and consistently structured according to the study objectives. The authors also appropriately include a section with the limitations of the study.

I only have a few recommendations.

Minor comments

The authors use the term "viral load" to refer to the Ct value obtained from the SARS-CoV-2 diagnosis assay, which is appropriate. However, the assay used to detect 10 respiratory viruses other than SARS-CoV-2 is qualitative and Ct values were not reported. Therefore, it is not correct to use the terms "viral load" or "viral burden" to refer to the number of viruses identified in coinfections. Both terms are quantitative and are normally used to report viral genome copies or the amount of infectious virus.

In Results there is a section entitled "Impact of viral burden"; however, the authors are not reporting viral loads, but the number of coinfecting viruses. This section should instead be named as "Impact of viral diversity" or "Impact of the number of co-detected viruses". Likewise, the term "viral burden" should be replaced throughout the manuscript by "coinfection burden" or "number of co-detected viruses" when referring to the number and type of viruses rather than viral quantity.

Finally, I suggest including a schematic representation or figure summarizing the multivariate analysis results to better illustrate the associations between viral co-detections (number and type) and clinical symptoms, thereby integrating the observed associations with severe disease.

a) Adequacy of the abstract: Yes

b) Originality and importance of the contribution for the development of the field of study: Yes

Relevance of:

c) Methodology, results and discussion: Yes

d) References: Enough

e) Figures and tables: Yes, although I suggest including a schematic representation or figure summarizing the multivariate analysis results.

## AUTHORS' RESPONSE TO THE REVIEWERS

**Response to Reviewer 1**

We thank the reviewer for the revision of our manuscript, and the answers to the questions are presented below. The corrections made in the manuscript are highlighted in the text.

**Question 1:**

"The authors mention removing variables with excessive missing data. Could you please specify the threshold used to justify variable exclusion? I also suggest including a brief description of the total number of records collected and the number excluded after data cleaning, to improve transparency and reproducibility."

*Author Response:*

We thank the reviewer for highlighting the need for greater transparency regarding our data cleaning and selection process. We have revised the Materials and Methods section to clarify the criteria used for variable exclusion and to provide a detailed description of the sample selection flow.

Regarding the threshold for missing data, we clarify that variables were excluded primarily based on statistical stability rather than a fixed percentage cutoff. Variables that exhibited insufficient variability, specifically those that became constant or had fewer than two levels after treating "unknown" responses as missing data, were excluded to ensure model convergence and stability. This information was included on page 8 of the revised and marked manuscript.

Regarding the sample description, we have included the total number of records initially retrieved and the number excluded to improve reproducibility. This information was included on page 7 of the revised and marked manuscript.

**Question 2:**

"Which continuous variables were categorized for the analysis? Additionally, regarding the adjustment for age groups, which age intervals were considered? Please specify these details in the Methods section."

*Author Response:*

We appreciate the opportunity to clarify the handling of continuous variables. In our study, continuous variables were treated differently depending on their role in the analysis.

Specifically, SARS-CoV-2 cycle threshold (Ct) values and time-related variables (such as days from symptom onset to sample collection) were categorized into quartiles for exploratory analysis and to assess non-linear associations. Age data were also processed into two categorical variables (ageYearsCat1: 1-2, 3-5, 6-11 years; ageYearsCat2: ≤5, > 5 years). Conversely, for the multivariate logistic regression models, age and the interval from symptom onset to sampling were retained as continuous variables to serve as adjustment confounders.

We have updated the Statistical Analysis section to explicitly state these procedures on page 8.

**Question 3:**

"On page 15, line 44: several of the severity symptoms described as closely associated are also used as classification criteria for SARI cases by the Brazilian Ministry of Health and the WHO. I believe that acknowledging this in the Discussion would enrich the contextualization of the findings."

*Author Response:*

We appreciate this important comment, and we have incorporated this contextualization into the Discussion on page 13 of the revised manuscript.

**Question 4:**

"I also suggest providing more detail in the figure and table legends, including the study setting and the period of data collection."

*Author Response:*

We revised all figure and table legends to include the study setting (Rio Grande do Norte, Northeast Brazil) and the study period (2021--2024), as suggested.

**Response to Reviewer 2**

We thank the reviewer for the careful assessment of our manuscript and for the constructive comments. We appreciate the positive evaluation of the study's relevance, structure, and methodological approach. We have carefully considered all suggestions and revised the manuscript accordingly, particularly regarding the terminology used to describe co-detected viruses and the inclusion of a graphical summary of the main findings. The corrections made in the manuscript are highlighted in the text. The schematic figure for a better description of the multivariate analyses was included as Figure 1.

# Reviewer report: REVIEWERS' COMMENTS

## REVIEWER #1

The authors of the revised manuscript, "Respiratory viral co-detection in pediatric SARS-CoV-2 infection: associations with viral co-circulation and clinical phenotypes" (ID: MIOC-2026-0121.R1), have adequately addressed the comments performed during the initial review. The changes are consistent with the recommendations provided in the minor comments and have improved the accuracy of the manuscript. After evaluating the revised version and the authors' responses, I consider that the manuscript is well organized and suitable for publication in its current version.

## REVIEWER #2

The paper is very relevant, original, well written, and all methodological gaps were clarified during the revision process. I have no further comments and recommend publishing the paper.
